# Health in the Irish Free State

**Published:** 1924-04

**Authors:** 


### HEALTH IN THE IRISH FREE STATE.

(From a Correspondent.)

In the Free State to-day supervision of the sanitary preparation of food is practically non-existent. Milk and other foods are frequently prepared and kept under appalling conditions of dirt, and the medical profession, recognising the amount of preventable disease this engenders, are determined to tackle the whole question of medical services for the people. Possibly one of the main reasons for the absence of sanitary science is the almost entire lack of active women's organisations. In the problems which most affect Irish homes and Irish children Irishwomen are taking very little interest.

### Doctors Growing Impatient.

Doctors, however, are becoming increasingly active, and at a recent meeting in the Royal College of Science, with Dr. Joseph Power, of Ardfinnan, in the chair, they came to some far-reaching decisions, the most important of which was to the effect that if the Government Commission of Inquiry was not satisfactory, or if the Dail should refuse to enact necessary legislation, the doctors will hold a delegates' meeting to decide as to the advisability of withdrawing from the public medical services. The Commission of Inquiry was promised by the Minister of Finance to Sir Joseph Glynn, and is to inquire into the working of the Health Insurance Acts in the Free State and into the whole question of the medical services. Medical men have now passed a resolution asking the Minister for Finance to have representatives of the profession, nominated by the Committee, on the Inquiry.

### Demands of the Medical Committee.

The Irish Medical Committee, the supreme body of the two medical organisations in the Free State, has passed a resolution strongly protesting against the reduction of the remuneration for medical certification under the Insurance Act and agreeing to accept the Irish Insurance Commission's terms only until August 31 next. It has also decided to make an immediate effort to organise all medical men in the Free State into a compact body and to make an immediate levy of £2 per head of the profession in Ireland to pay the expenses of an organiser. The Committee demands, moreover, that the scales of salaries adopted by the profession in Great Britain for whole-time Medical Officers, Tuberculosis Officers and Medical School Inspectors shall be applicable to Ireland.

### Primitive Conditions.

Medical Services are already being revised by the Government in some degree. They are, for example, abolishing workhouse hospitals for all chronic or serious cases of illness, which for the future will be transferred to the County Hospital under the charge of a special County Medical Officer. The workhouse infirmaries will henceforward accommodate only light and temporary cases of illness. The whole position of the medical care of the people is, however, as yet so imperfect that in parts of the country a patient has to be taken fifty miles in an ambulance to reach a hospital, and in some islands off the Western Coast a doctor visits the people only every few months. In the case of sudden illness it would be necessary to make an often dangerous journey to the mainland. All these matters have to be remedied as soon as the Free State finds time to look after her own internal problems.

### Sweepstakes for Hospitals.

The Sweepstake Bill, which was to permit the Free State hospitals to hold sweeps for the raising of funds, has for the moment been dropped. Rules for the introduction of private Bill legislation for the present session have not yet been completed. Meantime the funds of most of the Irish hospitals are in a very parlous state.

---

### STUDENTS AND EXTENSIONS.

An important series of improvements is being considered by the Queen's Hospital at Birmingham. As one of the leading schools connected with the University, the Queen's Hospital is anxious to keep pace with the growth of the number of students, its own need for further beds, and the claims of the special departments. It is therefore hoped to provide beds for maternity and tuberculous patients, and for cases of ear, nose and throat disease. There is an immediate desire to rebuild the surgical block, and to enlarge the out-patient department. The present buildings are, respectively, 83 and 51 years old. Last year, however, there was a deficit of over £5,000 on the working income, so that the financial problem is acute. Meantime, a plan is being considered to reserve a specified number of beds at the Blackwell Convalescent Home, whereby both the Home and the Hospital will benefit. This should help also to relieve the pressure on the wards. The obligations of a hospital which is also a medical school are always onerous, and indeed the needs of the students, of which the medical staff are keenly aware, help, by the vigilant demands of the latter, to keep the hospital up-to-date. It still remains unexplained, however, why Birmingham should continue to do less than its duty in supporting voluntary hospitals.

